# Immunogenicity of mammary tumor cells can be induced by shikonin via direct binding-interference with hnRNPA1

**DOI:** 10.18632/oncotarget.9660

**Published:** 2016-05-27

**Authors:** Shu-Yi Yin, Thomas Efferth, Feng-Yin Jian, Yung-Hsiang Chen, Chia-I Liu, Andrew H.J. Wang, Yet-Ran Chen, Pei-Wen Hsiao, Ning-Sun Yang

**Affiliations:** ^1^ Agricultural Biotechnology Research Center, Academia Sinica, Taipei, Taiwan, ROC; ^2^ Institute of Pharmacy and Biochemistry, University of Mainz, Germany; ^3^ School of Medical Laboratory Science and Biotechnology, Taipei Medical University, Taipei, Taiwan, ROC; ^4^ Institute of Biological Chemistry, Academia Sinica, Taipei, Taiwan, ROC

**Keywords:** shikonin, heterogeneous nuclear ribonucleoprotein A1, tumor immunogenicity, immunogenic cell death

## Abstract

Immunogenic cell death (ICD) of tumor cells occurs via various pathways that activate immune cell systems against cancer. Previous studies have demonstrated that shikonin (SK), a plant secondary metabolite, can confer strong pharmacological activities that activate ICD and strong immunogenicity of tumor cells. However, the exact hierarchical regulatory mechanisms including the molecular targets of SK-activated immunogenicity are still unknown. Here, the heterogeneous nuclear ribonucleoprotein A1 (hnRNPA1) was revealed to serve as a specific protein target for SK. This binding plays a key role in SK-stimulated ICD activity and the suppression of post-transcriptional mRNA processing, including nuclear export activity of newly synthesized mRNAs in mammary carcinoma cells *in vitro*. Moreover, it also mechanistically mediates the anti-metastatic effect of a tumor cell lysate (TCL) vaccine, which can be readily generated from SK-treated 4T1 tumor cells (SK-TCL), and the derived tumor-immunogenicity of SK-TCL-treated dendritic cells *in vivo*. Together, the identification of hnRNPA1 as the intracellular molecular target provides compelling pharmacology-based knowledge for the potential clinical use of SK-induced immunogenicity. In addition, SK may also serve as a potent suppressor that interferes with specific post-transcriptional activities, a mechanism which may be useful for exploitation in cancer therapeutics.

## INTRODUCTION

Immunogenic cell death (ICD) is a cell death modality that effectively stimulates an immune response against dying or dead cell antigens, in particular when they are derived from cancerous cells [1–3]. Recent studies in cancer immunotherapy have actively evaluated the use of patients' own dying tumor cells as a vaccine source to stimulate tumor-specific immune responses to control residual cancer cells, especially against metastatic malignant cells [4–6]. ICD of tumor cells and the derived TCLs have been shown to induce highly effective anti-tumor immunity in dendritic cells (DCs) and the consequent activation of specific T cell responses [7–11]. Such a DC-based therapeutic approach has been approved by the FDA for the treatment of specific prostate cancers [12]. We and others previously reported that shikonin (SK), a phytochemical derived from the medicinal plant *Lithospermum erythrorhizon* (LE), can serve as an efficacious adjuvant for expression of damage-associated molecular patterns (DAMPs) and the subsequent induction of ICD in treated carcinoma cells [3, 8]. Moreover, this SK-treated tumor cell lysate (SK-TCL) can be further employed to induce strong *in vivo* anti-tumor activity for a dendritic cell (DC)-based cancer vaccine [8, 11]. The identification of hierarchical regulatory mechanisms of SK will be necessary and important for future clinical application of the SK-induced cellular ICD in development of cancer immunotherapy.

Killer lymphocytes are known to induce ICD in targeted cells via the deployment of cytotoxic granule serine proteases, such as granzymes A (GzmA) and GzmB [13, 14]. hnRNPA1 has been shown to be an important GzmA substrate that can impair the nuclear export of newly synthesized RNA and disrupt pre-mRNA splicing [15]. Such alterations in RNA processing result in a specific type of immune-mediated programmed cell death [15]. In the present study, we found that the interaction between SK and hnRNPA1 was able to directly suppress the nuclear export activity of newly synthesized RNA and the binding of a defined nucleotide sequence recognized by hnRNPA1. These findings demonstrate the importance of hnRNPA1 in the activity of SK-induced ICD in tumor cells. In contrast with many other ICD inducers, such as doxorubicin and mitoxantrone [3, 16], we show here that SK can induce tumor cell ICD without genetic modification. This suggests that SK may be a potentially useful experimental or clinical medicine for stimulating specific hnRNP-mediated ICD.

Over the past three decades, there have been a large number of studies (thousands of research papers reported and over 500 patents issued) on shikonin and its derivatives. We showed previously that SK can confer a broad spectrum of biochemical activities, including the inhibition of promoter and RNA splicing activities of TNF-α [17, 18] and GM-CSF [19], the induction of epithelial-to-mesenchymal transition (EMT) activity in skin wound-healing [20], and others [21]. Due to this broad spectrum of biochemical activities, SK has been actively investigated for potential application in the treatment of various inflammatory diseases [22–24]. In related studies, SK and its analogs have also been indicated to be potent inhibitors of a tumor-specific pyruvate kinase-M2 (PKM2) [25], a potential molecular target for disrupting glucose metabolism in cancer cells [26, 27]. In this study, we identified another molecular target of SK, namely heterogeneous nuclear ribonucleoprotein A1 (hnRNPA1), which is known to play a key role in lymphocyte-induced ICD in targeted cells [3, 13, 14, 23]. We believe this study provides important molecular targeting and cellular evidence to support the multi-faceted pharmacological activities reported for SK, including its anti-inflammatory, anti-cancer and wound-healing activities.

In the present study, molecular docking and in silico virtual screening software were used to search for candidate molecular targets of SK. By using a combination of antibody pull-down assay and MS/MS analysis, we further biochemically confirmed the binding activity between SK and the hnRNPA1 protein in SK-treated human mammary cancer cells (MDA-MB-231). Specifically, the disruption of hnRNPA1 function is necessary for the expression/translocation of ICD markers in SK-treated tumor cells. The *in vivo* anti-metastatic effect of tumor cell lysate (TCL) and the derived TCL-pulsed DC vaccine further supports the critical role of hnRNPA1 in the immunogenicity of TCL resulting from stimulation by SK. Via binding to hnRNPA1, SK was also shown to be an effective suppressor of specific post-transcriptional and ICD effects that promote tumor-immunogenicity of treated tumor cells.

## RESULTS

### hnRNPA1 is a mammalian intracellular target of shikonin

**Figure 1:**
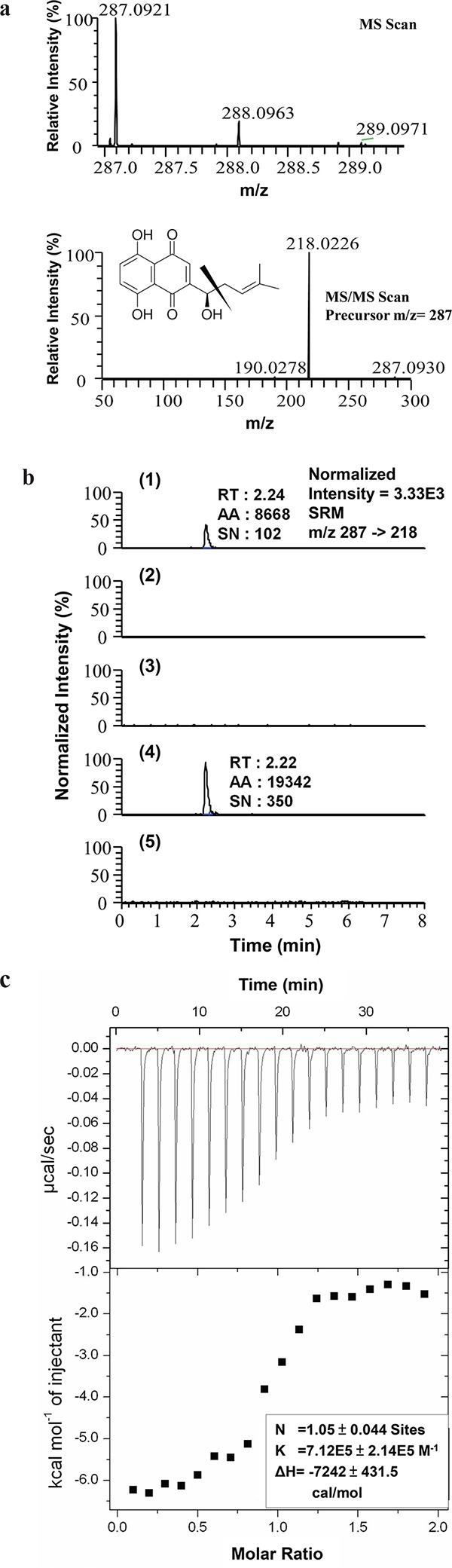
Evaluation of hnRNPA1 as an intracellular target of SK **a.** MS and MS/MS scan of SK standard. The acquisition range for SK detection is between 215.5 to 220.5 m/z; **b.** LC-MS/MS analysis of (1) SK standard (10 pg) dissolved in methanol; (2) the sample prepared from the anti-hnRNPA1 antibody-conjugated magnetic beads alone and dissolved in methanol without mixing with any TCL (vehicle control group); (3) hnRNPA1 molecules pull-downed from TCL derived from SK-untreated MDA-MB-231 cells; (4) hnRNPA1 molecules pulled down from TCL derived from SK-treated MDA-MB-231 cells; and (5) Actin molecules pulled down from TCL collected from SK-treated MDA-MB-231 cells, by using anti-Actin antibody-conjugated beads. Images are representative of at least three different experiments. RT, retention time; AA, peak area; SN, signal to noise. **c.** Isothermal titration calorimetry (ITC). Representative isotherm from SK: hnRNPA1 (UP1) titrations at pH 7.0. 300 μM SK titrated against 14 μM UP1 until saturation, suggesting a single binding site with molar ratio of 1 (N = 1.05 ± 0.044 site).

**Figure 2:**
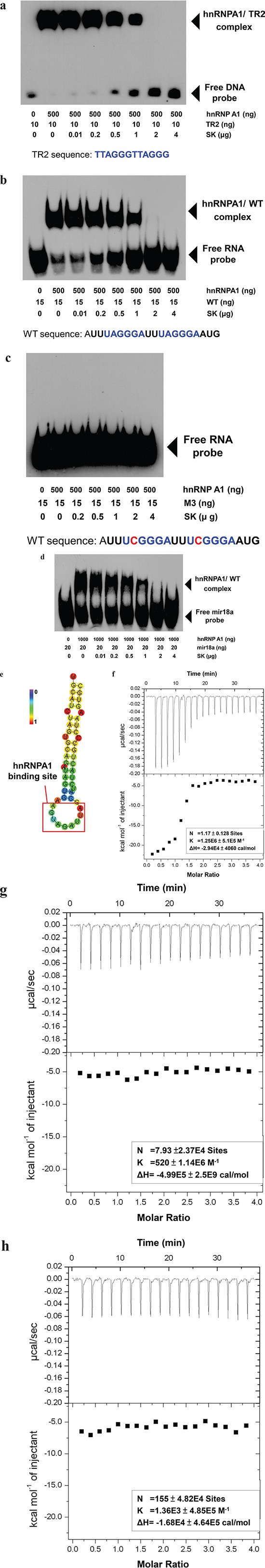
SK suppresses the recognition activity of hnRNPA1 of a targeted nucleotide sequence and the splicing activity of hnRNPA1-targeted genes *in vitro* **a.** The recombinant human hnRNPA1 protein (UP1) samples produced from an *E. coli* pET-42a expression system were prepared at 0.5 μg/20 μl and then incubated with increasing amounts of SK (0-4 μg/test) for 30 min. The mixture was then incubated with biotin-5′-end-labeled TR2 DNA (10 ng/test) for another 30 min. The samples were loaded on a 5% native polyacrylamide gel for EMSA. **b.** The purified UP1 was incubated with increasing amounts of SK (0-4 μg/test) for 30 min and then incubated with biotin-5′-end-labeled WT RNA (50 ng/test) for another 30 min. **c.** UP1 samples were incubated with increasing amounts of SK and then with biotin-5′-end-labeled M2 RNA (15 ng/test) in which the recognition sequence was changed at one specific base (A→C). **d.** UP1 (1 μg/20 μl) was incubated with increasing amounts of SK and then with synthesized biotin-5′-end-labeled microRNA-18a (mir18a; 15ng/test). All UP1-DNA and UP1-RNA complex samples were subjected to *LightShift* Chemiluminescent *EMSA*. **e.** The hairpin structure of the sequence for miR-18a was predicted by RNAfold Web Server. Bold red square, recognition site for bound hnRNP A1. The structure is colored by base-pairing probabilities, according to left-node bar. Data are representative of two or more independent experiments. **f.** Isothermal titration calorimetry (ITC). Representative isotherm data from RNA (WT): hnRNPA1 (UP1) titrations at pH 7.0. 60 μM WT RNA was used to titrate against 3 μM UP1 until saturation, the data suggests a single binding site with a molar ratio of 1 (N = 1.17 ± 0.128 site). **g.** Displacement isothermal titration calorimetry. Prior to ITC analysis, 3 μM UP1 protein was mixed with 60 μM WT RNA for 30 min. UP1/RNA samples were then titrated by 60 μM SK, indicating that SK did not show significant binding activity to WT RNA-mixed UP1 molecules. **h.** The WT RNA: SK titration data was attached as a negative control test. The sample of SK (60 μM) titrated against WT RNA (60 μM) did not show significant binding of SK to WT RNA molecules.

**Figure 3:**
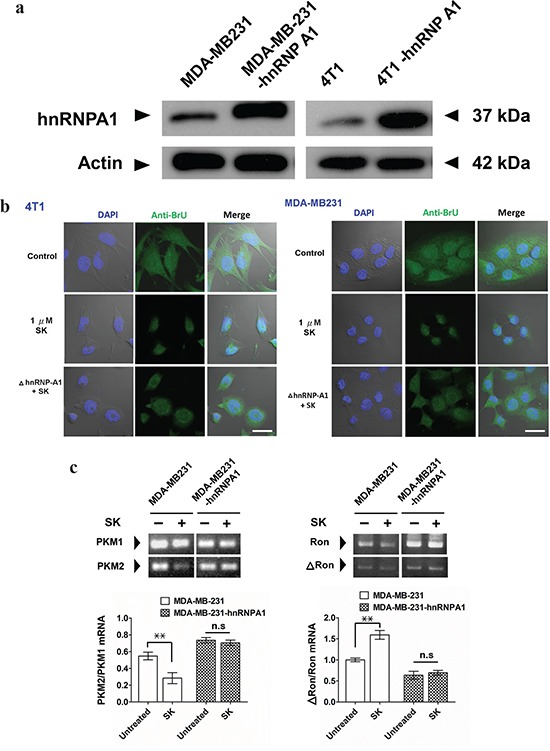
SK treatment disrupts the hnRNPA1-mediated nuclear export activity of newly synthesized RNA **a.** Western blot analysis for expression of hnRNPA1 in 4T1, 4T1-hnRNPA1, MDA-MB-231 and MB-231-hnRNPA1 cells. Beta-actin staining was used as a loading control. **b.** Immunofluorescent staining for detecting newly synthesized RNA. In some groups, SK was treated at 1 μg/ml for 2 h. All test cells were then incubated with 2 mM bromouridine for another 2 h at room temperature. Anti-BrU antibody conjugated with FITC was used for detecting newly synthesized RNA. DAPI was used to stain the nucleus. Scale bars, 20 μm. Data are representative of two or more independent experiments. **c.** RT-PCR analysis for expression ratio of *PKM1*/*PKM2*. RNAs were extracted from control, SK-treated and Dox-treated MDA-MD-231, as well as MDA-MD-231-hnRNPA1 cells, which were normalized to the expression level of an endogenous reference gene *GAPDH*; *n* = 3 cell samples per group; **, *P* < 0.01. **d.** RT-PCR analysis for expression ratio of Δ*RON*/*RON*; *n* = 3 per group. Error bars represent SD. **, *P* < 0.01. P values were calculated using Student's *t* test. Data are representative of three independent experiments.

**Figure 4:**
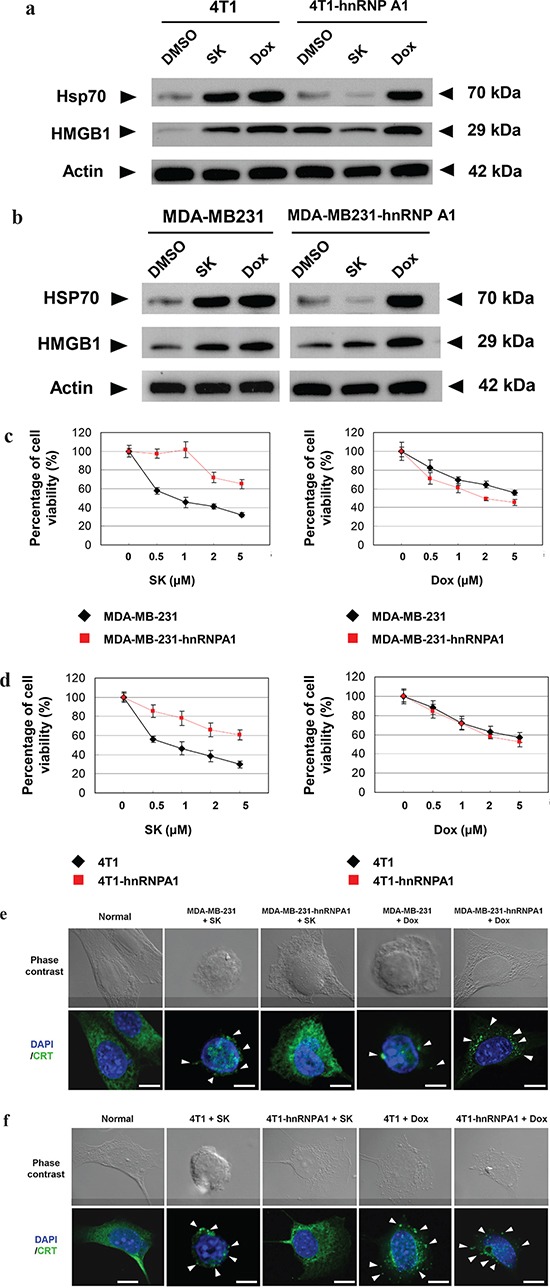
hnRNPA1 is a critical mediator of the SK-induced ICD activity in mammary tumor cells **a.** and **b.** Western blot analyses of expression of HSP70 and HMGB1 in human (MDA-MB-231) and mouse (4T1) tumor cells. Some 4T1, 4T1-hnRNPA1, MDA-MB-231 and MB-231-hnRNPA1 cells were treated with SK or Dox at 5 μg/ml for 24 h. β-actin was used as a loading control. **c.** and **d.** MTT assays to test the cytotoxicity of SK and Dox on various tumor cells. 4T1, 4T1-hnRNPA1, MDA-MD-231 or MDA-MD-231-hnRNPA1 cells were dispensed in 96-well plates (1 × 10^4^cells/well) and incubated with vehicle, SK or Dox for 24 h. All treatments were performed in triplicate cultures. **e.** and **f.** IF staining for localization of endogenous CRT in mammary tumor cells. 4T1, 4T1-hnRNPA1, MDA-MD-231 or MDA-MD-231-hnRNPA1 cells were treated with vehicle, SK or Dox for 24 h before staining for CRT (green) and DAPI (blue). Scale bars, 10 μm. Data are representative of two or three independent experiments.

**Figure 5:**
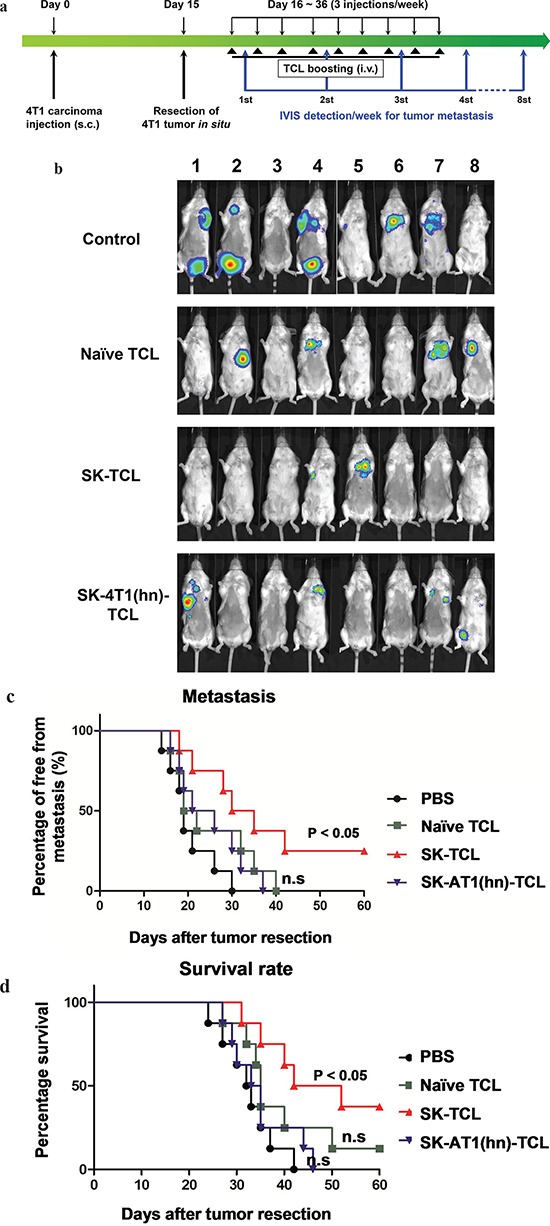
Anti-metastatic effect of SK-treated tumor cell lysate is mediated by the disruption of hnRNPA1 function **a.** Schema of experimental design. BALB/c mice were injected with 4T1-Luc2 cells (5 × 10^5^ cells/mouse) into mammary fat pad. At 15 days post tumor cell implantation, primary 4T1 tumors were resected and mice were then administered with PBS or different TCL samples (100 μg/200 μl/mouse) for 3 weeks (3 injections/week) post tumor resection. Mice were compared for their tumor metastatic activity (using IVIS imaging system) and survival rate. **b.** Representative bioluminescent images of 4T1-Luc2 tumor-resected mice at 3 weeks post tumor resection. Mice in different groups were treated *in vivo* with control (1 × PBS), 100 μg naïve TCL, SK-TCL or SK-4T1(hn)-TCL at 0, 1 and 2 weeks post tumor resection (1 injection/week). The red signal represents the highest level on the colorimetric scale. **c.** Quantification of tumor metastasis by measuring luciferase activity in p/s/cm^2^/sr in mice revealed along the indicated time points. **d.** Survival of test mice after different treatments. *n* = 8 per group. P values were calculated by using a log-rank (Mantel-Cox) test of the Kaplan-Meier survival curves. *, *P* < 0.05. n.s, not significant. Data are representative of three independent experiments.

To search for molecular targets of SK in mammalian cells, we first conducted a bioinformatics prediction analysis using a molecular docking and virtual screening system. In a comparison of the putative molecular affinity between SK and more than 27,000 human protein or peptides, hnRNPA1 was predicted to exhibit the highest binding/docking affinity with SK (Table 1). To verify this possible molecular binding activity, an antibody pull-down assay in combination with LC-MS/MS analysis was employed to determine the intracellular interaction between SK and hnRNPA1 protein. The chromatographic and mass spectrometric conditions for detection of SK in this assay were optimized from a previous report [28]. The production spectrum of an SK standard (Figure 1a) showed a significant precursor ion signal (m/z = 287.0921) and the MS/MS fragmental spectra for its precursor showed a major fragment (m/z = 218.0226). In LC-MS/MS spectra, the retention time (RT) for the SK standard was 2.24 min (Figure 1b-1). We then analyzed the presence of SK molecules associated with intracellular hnRNPA1 protein, which was pulled down from the crude protein extract of human mammary tumor cells (MDA-MB-231), using anti-hnRNPA1 antibody-conjugated magnetic beads. Based on this approach, in SK-treated cells once enough SK molecules interacted with intracellular hnRNPA1, the associated SK molecules could be dissociated from the antibody-protein complex, and became detectable by MS/MS analysis as described above. For the vehicle control group, only the anti-hnRNPA1 antibody-conjugated magnetic beads without incubation with tumor cell extract, were dissolved in methanol, hence the control group did not show a corresponding peak for SK at specific RT in MS/MS chromatography (Figure 1b-2). Similarly, the SK-untreated cells also did not exhibit the corresponding peak for SK (Figure 1b-3), showing that the pulled down hnRNPA1 were not associated with SK molecules. In comparison, only the test sample from SK-treated MDA-MB-231 cells (Figure 1b-4) showed a major fragment (m/z = 218.0226) for SK at the expected retention time (RT = 2.22 min). To assess the specificity of such SK-hnRNPA1 binding, antibodies conjugated with anti-β-Actin (Figure 1b-5), anti-α-tubulin and anti-hnRNPA2 antibodies (data not shown) were also used to pull down specific proteins in SK-treated MDA-MB-231 cell extracts. Again, the corresponding fragment of SK was not detected in these negative control samples. Together, these results suggest that hnRNPA1 is indeed a specific intracellular protein target of the phytochemical SK.

**Table 1:** Ranking of selected SK-interacting protein candidates

Number	Protein Name	Binding energy [kcal/mol]
1	Heterogeneous nuclear ribonucleoprotein A1	−15,3
2	Ig gamma-1 chain C region (secreted form)	−12,0
3	Cell division protein kinase 2 (CDK2)	−11,5
4	Aldose reductase	−11,4
5	Alpha parvin	−10,7
:		:
27316	Collagen alpha-1(III) chain	3.4
27317	Discoidin domain-containing receptor 2	3.4

Isothermal titration calorimetry (ITC) analysis was subsequently performed to determine the thermodynamics of the interaction between hnRNPA1 and SK molecules. Consistently, SK was found to bind to hnRNPA1 protein with a K_d_ value of 71.2 μM on average (Figure 1c), with an experimental stoichiometry value close to 1 (N = 1.05 ± 0.044 site). In agreement with the pull-down assay data (Figure 1a and 1b), this result confirms the direct binding relationship between SK and hnRNPA1.

### SK directly suppresses the recognition of a defined nucleotide sequence by hnRNPA1

To evaluate the specific effect of SK on the post-transcriptional functions of hnRNPA1, we tested the *in vitro* effect of SK on the interaction between the N-terminal fragment of a recombinant hnRNPA1 protein (UP1) and a defined recognition DNA (TR2) or RNA (WT) sequence [29, 30], which was used to evaluate the specific binding activity between a hnRNPA1 pocket and specific recognition nucleotide sequence. Using EMSA, we found that SK was able to directly disrupt the binding between hnRNPA1 and TR2 in a dose dependent manner (Figure 2a). Consistently, SK exhibited a similar effect on the binding between hnRNPA1 and a single-stranded RNA (WT sequence: 5′-AUUUAGGGAUUUAGGGAAUG-3′) (Figure 2b), which has also been previously defined to confer efficient binding to hnRNPA1 [31]. In contrast, when this RNA sequence was changed into the M3 sequence, i.e., 5′-AUUUCGGGAUUUCGGGAAUG-3′ (M3: with one A→C nucleotide mutation), it could no longer be recognized by the UP1 [32]. We show here that M3 can be used as a negative control for the specificity of nucleotide recognition with the hnRNPA1 binding (Figure 2c). Moreover, we analyzed the suppressive effect of SK on the recognition activity of hnRNPA1 on miR-18a (Figure 2e), which has been identified as a specific microRNA target of hnRNPA1 [33]. In support of the specific binding activity of SK on hnRNPA1 protein, SK can also suppress the recognition of hnRNPA1 to a specific microRNA structure (Figure 2d). Taken together, these results suggest that SK can efficiently suppress hnRNPA1 targeting of specific nucleotides with defined sequences or secondary structures.

Using ITC analysis, we further evaluated whether SK could affect the RNA-binding to UP1 molecules. WT RNA was found to bind to UP1 (Figure 2f) with an experimental stoichiometry of close to 1 (N = 1.17 ± 0.128 site). Once UP1 molecules were pre-treated with WT RNA, SK could no longer effectively confer significant binding to UP1 in ITC analysis (Figure 2g). Hence, SK can interact specifically with the naïve hnRNPA1 molecule (without RNA binding), rather than act on the RNA-bound hnRNPA1 molecules. To exclude the possibility that the SK molecule can bind to the naked RNA by itself, a stand-alone SK control was also titrated against the WT RNA, and no significant binding was detected (Figure 2h).

### SK disrupts the hnRNPA1-mediated nuclear export activity of newly synthesized RNA and the splicing activity of hnRNPA1-targeted genes

To further investigate the role of hnRNPA1 in SK-induced post-transcriptional regulation, we overexpressed the human and mouse hnRNPA1 in human MDA-MB-231 and mouse 4T1 mammary tumor cells, respectively. Transgenic MDA-MB-231 cells (MDA-MB-231-hnRNPA1) expressed a much higher level of hnRNPA1 than the parent MDA-MB-231 cells (Figure 3a). In addition, transgenic 4T1-hnRNPA1 cells overexpressing hnRNPA1 were also established (Figure 3a). Immunofluorescence staining with anti-BrU antibody was used to assess the effect of SK on nuclear export activity of newly synthesized BrU-labeled RNA in human and mouse mammary tumor cells (Figure 3b). In comparison, wild-type MDA-MB-231 and 4T1 cells treated with SK showed much lower nuclear export activity of newly synthesized RNA than the untreated counterpart cells. In contrast, overexpression of hnRNPA1 in test cells was able to rescue this SK-induced suppression of the RNA nuclear export activity (Figure 3b). These results reveal that hnRNPA1 has an important role in the SK-induced regulation of specific RNA processing.

We next used RT-PCR analysis to assess the *in vitro* regulatory effect of SK on the splicing of hnRNPA1-targeted genes, including pre-mRNA encoding pyruvate kinase (PKM) [34–36] and tyrosine kinase receptor (Ron) [37, 38]. *In vitro* treatment of MDA-MB-231 cells with 1 μM SK downregulated the expression of PKM2, as revealed by the ratio of *PKM2/PKM1* detected (Figure 3c). In contrast, the expression ratio of *PKM2/PKM1 in MDA-MB-231-hnRNPA1 cells was not significantly changed after* SK treatment (Figure 3c). On the other hand, SK treatment significantly increased the expression ratio of *ΔRon/Ron* in MDA-MB-231 cells (Figure 3d). The ΔRon is a constitutively active isoform of the Ron tyrosine kinase receptor, arising from skipping the splicing of Ron exon 11 [38, 39]. Similarly, the same SK treatment did not significantly change the expression ratio of *ΔRon/Ron* in MDA-MB-231-hnRNPA1 cells (Figure 3d). These results therefore suggest that SK can mediate disruption of hnRNPA1 functions and further regulate the splicing activities of hnRNPA1-targeted genes.

### hnRNPA1 is a critical mediator of SK-induced immunogenic cell death

To investigate whether SK-induced ICD is via the suppression of hnRNPA1 activity, we compared the change in expression patterns of specific ICD markers, including HSP70 and HMGB1, in response to SK treatment in tumor cells. Since doxorubicin (Dox) has been shown to stimulate ICD [16], it was used as a positive control for ICD induction. For human MDA-MB-231 cells, both SK and Dox treatments at 5 μM for 24 h significantly increased the expression of HSP70 and HMGB1 (Figure 4a). However, when these cells were transfected to overexpress hnRNPA1 proteins (i.e., MDA-MB-231-hnRNPA1) and then treated with SK or Dox, test cells responded only to Dox treatment, but not to SK treatment. Similar results (Figure 4b) were also consistently observed for mouse 4T1 cells versus 4T1-hnRNPA1 cells, in response to SK and Dox treatments. These data strongly suggest that hnRNPA1 can act as an important mediator of the phenotypic expression of SK-induced ICD.

To determine whether hnRNPA1 dysfunction plays an essential role during SK-induced cell death, MDA-MB-231-hnRNPA1 cells were treated with SK or Dox, and cell viability was evaluated by MTT assay. Overexpression of hnRNPA1 efficiently rendered test cells much more resistant to SK treatment than untransfected cells, but these two cell types were equally sensitive to Dox treatment (Figure 4c). Similar results were also observed between mouse 4T1 and 4T1-hnRNPA1 cells (Figure 4d). We therefore conclude that hnRNPA1 can serve as an important mediator of SK-induced cell death.

One key feature of the tumor cell ICD is the appearance of surface-exposed calreticulin (CRT) on the cell membrane [16, 40]. To further investigate whether hnRNPA1 can also play a role in the surface-exposure of CRT in response to SK, immunofluorescence staining was used to detect the intracellular distribution of CRT. The distribution of intracellular CRT in untreated MDA-MB-231 cells was found in the cytosol, presented as fibrillar and punctuate structures. In comparison, in SK-treated cells CRT proteins were found to become much less abundant, and were observed as “patch like” structures (Figure 4e). A similar effect was also detected in the Dox-treated MDA-MB-231 cells. On the other hand, the MDA-MB-231-hnRNPA1 cells were highly resistant to this SK effect on CRT distribution, virtually restoring to the normal cell phenotype. This resistant phenotype was certainly not detected after Dox treatment (Figure 4e). Similarly, overexpression of hnRNPA1 in 4T1 tumor cells also reversed the protein translocation process of CRT molecules in response to SK (Figure 4f). These results suggest that the SK-induced distributional change of CRT proteins during ICD of tumor cells is also mediated by hnRNPA1. However, the Dox-induced CRT redistribution is obviously not dependent on the hnRNPA1 dysfunction.

### SK-induced disruption of hnRNPA1 function is required for the anti-metastatic effect of SK-treated tumor cell lysate

Tumor cell lysate (TCL) has the advantage of containing an extensive repertoire of tumor cell antigens, but it often requires adjuvants to enhance its immunogenicity when used as a tumor vaccine [41, 42]. In this study, we evaluated the anti-metastatic effect of SK-treated 4T1 tumor cell lysate (SK-TCL) on a mouse mammary carcinoma system mechanistically. Test 4T1 cells were treated with 5 μM SK for 24 h, and the derived SK-TCL sample was then compared for its anti-metastatic activities against 4T1-Luc2 tumors in a tumor resection model (see Materials and Methods). After tumor resection, the tumor metastatic activity and survival rate of mice treated with 1× PBS, naïve TCL (extracted from untreated 4T1 cells), SK-TCL (extracted from SK-treated 4T1 cells) and SK-4T1(hn)-TCL (extracted from SK-treated 4T1-hnRNPA1 cells) were compared (Figure 5a). By detecting luminescent activity in 4T1-Luc2 cells at 3 weeks post tumor resection, *in vivo* administration of SK-TCL (100 μg/mice) was found to significantly suppress the metastasis of 4T1-Luc2 cells into the lung (Figure 5b). In contrast, SK-4T1(hn)-TCL treatment showed only limited suppression of tumor metastasis as compared with naïve-TCL administration. As shown in Figure 5c, SK-TCL conferred much higher anti-metastatic activity than that obtained from treatment with naïve-TCL. Consistently, overexpression of hnRNPA1 in 4T1 cells (SK-4T1(hn)-TCL) also reversed the increase in survival rate of the SK-TCL-treated mice (Figure 5d). Together, our results show that antigenic immunity derived from SK-TCL can generate highly potent prevention of metastasis of 4T1 tumor cells. Furthermore, hnRNPA1 disruption plays a critical role in the anti-metastatic activity of SK-TCL.

### Disruption of hnRNPA1 function plays a key role in the anti-metastatic immunity of SK-TCL-activated dendritic cells

**Figure 6:**
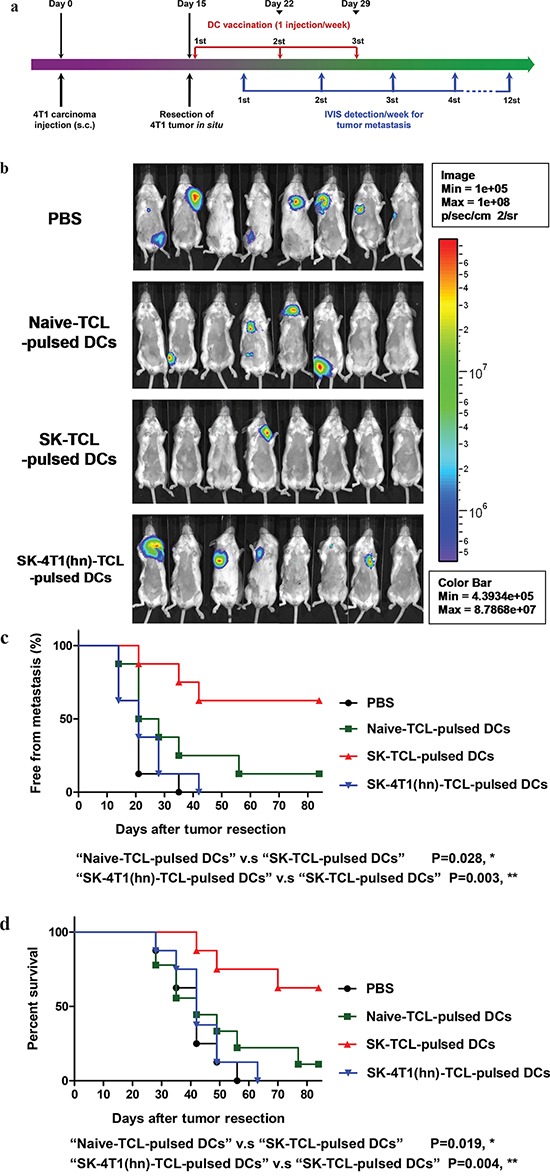
Anti-metastatic effect of SK-TCL-activated DC vaccines is mediated by the disruption of hnRNPA1 function **a.** Schema of experimental design. BALB/c mice were injected with 4T1-Luc2 cells (5 × 10^5^ cells/mouse) into the mammary fat pad. Primary 4T1 tumors grown for 15 days were resected surgically and test mice were then administered with PBS or different TCL-pulsed DC vaccines (1 × 10^6^ DCs/mouse) for 3 weeks (1 injection/week). Mice were then compared for their tumor metastatic activity (using IVIS imaging system) and animal survival rate. **b.** Representative bioluminescent images of 4T1-Luc2 tumor-resected mice at 3 weeks post tumor resection. The naïve-TCL, SK-TCL or SK-4T1(hn)-TCL were used to treat DCs for 24 h *in vitro*. Mice in different groups (n = 8) were then injected intravenously (i.v.) with control (1 × PBS), naïve TCL-pulsed DCs, SK-TCL-pulsed DCs or SK-4T1(hn)-TCL-pulsed DCs at 0, 1 and 2 weeks post tumor resection (1 injection/week). Bar graphs to the right indicating the quantification of luminescent activity in the mice. **c.** Quantification of tumor metastasis burden in mice treated within the indicated time course as revealed by bioluminescence imaging. **d.** Survival of test mice after different treatments. P values were calculated by using a log-rank (Mantel-Cox) test of the Kaplan-Meier survival curves. *, *P* < 0.05; **, *P* < 0.01, were obtained between each indicated group. Data are representative of three independent experiments.

Previously, a DC vaccine pulsed with SK-TCL was shown to effectively elicit a strong therapeutic antitumor immunity *in vivo* [3, 8]. We therefore further evaluated the role of hnRNPA1 in antitumor immunity of SK-TCL-activated dendritic cells. Similarly, 4T1 cells or 4T1-hnRNPA1 cells were treated with 5 μM SK for 24 h, and the derived SK-TCL or SK-4T1(hn)-TCL samples were then pulsed with test bone marrow-derived DCs (BMDCs). Different TCL-pulsed BMDC preparations were then compared for their anti-metastatic activity in the 4T1 mammary tumor-resection model (see Materials and Methods). After tumor resection, mice were injected with 1× PBS, naïve TCL-pulsed DCs, SK-TCL-pulsed DCs or SK-4T1(hn)-TCL-pulsed DCs, and the tumor metastatic activity and survival rate were compared between each group (Figure 6a). By tracking tumor metastasis for 12 weeks, *in vivo* administration of SK-TCL-pulsed DCs (1 × 10^6^ DCs/mice) was found to more effectively suppress the metastasis rate of 4T1-Luc2 cells into the lung, as compared with that in mice administrated with naïve TCL-pulsed DCs (Figure 6b and 6c). In contrast, mice vaccinated with SK-4T1(hn)-TCL-pulsed DCs exhibited only a similar level of anti-metastatic activity to that observed for the naïve TCL-pulsed DCs treatment. Consistently, overexpression of hnRNPA1 in 4T1 cells (SK-4T1(hn)-TCL) also reversed the tumor immunogenicity of TCL-pulsed DCs (SK-4T1(hn)-TCL-pulsed DCs) and the treatment decreased the survival rate of test mice, as compared with mice treated with SK-TCL-pulsed DCs (Figure 6d). These results together demonstrate that hnRNPA1 function can play a critical role in conferring the anti-metastatic activity of SK-TCL-activated DC vaccine.

### Computer modeling analysis of hnRNPA1/SK complex reveals two candidate binding sites for SK on hnRNPA1 protein

**Figure 7:**
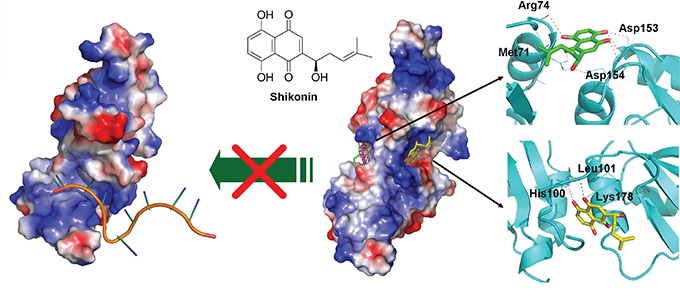
Molecular and structural model of the SK activity in blockade and regulating nucleotide-binding activity of hnRNPA1 Docking models of the SK binding to hnRNPA1, generated by AutoDock Vina and presented by a molecular visualization program, PyMOL. On the left, electrostatic surface of hnRNPA1 in complex with single-stranded (ss) DNA are shown (PDB ID: 1U1O). The ssDNA is indicated in orange. On the right, the two predicted sites for SK binding are presented (sticks with yellow or green carbon atoms). Amino acid residues that contribute to this binding stabilization are indicated with dotted lines.

With future demand for clinical applications of SK-induced ICD in tumor cells for development of cancer vaccines, next we explored the pharmacological mechanisms by which SK may target the human hnRNPA1/SK complex. The defined binding/interaction between the hnRNPA1 protein (UP1) and the recognized nucleotide sequence (TTAGGGTTAG) [43] is shown in Figure 7 (left panel). For post-transcriptional processing, this region of the UP1 also has a high affinity for single-stranded RNA [44, 45]. The hnRNPA1/SK complex as predicted by molecular docking analysis is shown in the right panel (Figure 7). The SK molecules shown in green, yellow and pink indicate the top three binding sites in preference/affinity, according to their calculated binding energy. The hnRNPA1 protein molecule is proposed to render a twisted conformation after binding to SK, and this configuration may further regulate the structure of RNA recognition motifs (RRMs) in hnRNPA1 and further disturb the hnRNPA1 function at the post-transcriptional level.

## DISCUSSION

Although shikonin has been shown to strongly stimulate the induction of tumor immunogenicity [3, 8, 46], the molecular mode of action and the pharmacological mechanism of this stimulatory effect are still controversial. In a previous study, the pyruvate kinase-M2 (PKM2) was indicated as a potent target of SK and its analogs [25]. Interestingly and in relevance to our present study, the alternative splicing between PKM1 and PKM2 has also been shown to be regulated by hnRNPA1 activity [35, 36, 47]. In our present study, the proposed target for SK binding, hnRNPA1, suggests that SK may regulate PKM splicing through an indirect mechanism. In contrast to mitoxantrone, doxorubicin, UVC irradiation, anthracyclines and others, which induce ICD by DNA-targeting or collateral ER stress effects [3, 48–52], SK is shown here to induce ICD in tumor cells at the post-transcriptional level. In view of these findings, we consider that ICD induced by SK without genetic interference may be a useful strategy for future development of cancer vaccines and immunotherapy. Other ICD inducers, such as oxaliplatin (OXP), have also been shown to induce ICD in colorectal cancer cells [53]. However, OXP was not able to induce DC activation and antigen presentation [54]. Based on these findings, we therefore also considered the possibility that the dysfunction of hnRNPA1 may play a key role in generation of tumor immunogenicity. To evaluate the potential universality and importance of hnRNPA1-targeting in these compound-induced ICDs, we also used a siRNA approach to knockdown the hnRNPA1 expression in 4T1 mammary carcinoma cells. Western blot analyses was used to measure expression of ICD markers, including HSP70, CRT and HMGB1, in SK- or OXP-treated mammary carcinoma cells (Supplementary Figure S1). Treatment with hnRNPA1 siRNA can efficiently knock down the expression level of hnRNPA1 (39 kDa) in 4T1 cells. The knockdown of hnRNPA1 resulted in a higher expression level of HSP70 and HMGB1 in 4T1 cells without SK or OXP treatment, as compared with untreated (Vehicle) or negative control siRNA-treated cells. This result confirmed the role of hnRNPA1 dysfunction in immunogenic cell death [15]. For 4T1 cells with a knockdown on hnRNPA1 expression, SK treatment did not significantly increase the expression levels of HSP70 and HMGB1. In contrast, the SK-increased CRT expression was still observed in the hnRNPA1-knockdown 4T1 cells (Supplementary Figure S1). This result indicates that hnRNPA1 may participate in specific, rather than all, SK-induced ICD marker expression. As compared with SK treatment, OXP treatment also considerably increased the expression level of HSP70 in 4T1 cells. The knockdown of hnRNPA1 did not change the OXP effects on ICD marker expression in 4T1 cells (Supplementary Figure S1). Together, these supplementary data further suggest that hnRNPA1 plays an important role in exposure of specific ICD features. Moreover, by considering the apparently non-essential role of hnRNPA1 in OXP-induced ICD activities, our results also suggest that hnRNPA1-targeting may not be universal for all ICD inducers with stimulatory activity on the ICD of tumor cells.

In normal somatic tissues, epithelial-to-mesenchymal transition (EMT) activity is not only instrumental in wound-healing but also in tissue fibrosis [55–57]. hnRNPA1 has been shown to play an important role in controlling the specific splicing activity of tyrosine kinase receptor (Ron), and can thus further promote the mesenchymal-to-epithelial transition (MET) activity [37]. Recently, we reported that topical treatment of mouse skin with SK can stimulate EMT and suppress the expression of the associated microRNAs during skin wound-healing [20]. Taking these findings together, we hypothesize that SK-induced EMT *in vivo* may also be due to dysfunction of hnRNPA1. In the future, it will be important to assess this pharmacological activity of SK in promoting tissue wound-healing and regulating specific hnRNPA1-target microRNAs.

In our previous study of a dendritic cell (DC)-based cancer vaccine, SK-TCL was found to confer a potent *in vitro* and *in vivo* adjuvant effect on activation of vaccinating DCs [8]. In this study, we show that *in vivo* administration of SK-TCL-pulsed DCs, and to some extent even the naïve-TCL-pulsed DCs, can significantly suppress the metastasis of 4T1 mammary carcinoma cells in a tumor resection model. These results suggest that, in order to be attacked by “self-immunity”, tumor cells need to be reprogrammed by specific “effector” components, such as HSP70, HMGB1 and CRT, resulting in activating vaccinated DCs *in vitro* or enhancing tumor immunogenicity *in vivo*. In future study, it will be necessary to evaluate each of these components in the form of SK-induced TCL and to optimize the possible synergistic effect on prevention of tumor metastasis.

Previously, the administration of tumor cell lysate (TCL) or DC vaccines has been mainly performed via subcutaneous or intravenous injection, including injection into the footpad of test animals [3, 8, 11, 41, 42]. In the 4T1 mammary carcinoma system, the primary metastatic target organs are known to be the lung and spleen. We intended to maximize the anti-metastatic effects of the shikonin-induced ICD preparations of tumor cell vaccines by cutting down the tissue travelling barrier or/and time-span for test TCL or DCs preparations to reach the lung and spleen tissues. Intravenously injected DCs have been previously shown to result in good distribution into the lungs, liver and spleen, whereas subcutaneously injected DCs migrated primarily to the draining lymph nodes [58]. Intravenous administration of DC vaccine has also been employed in recent clinical trials to treat advanced non-small cell lung cancer [59]. Consistently, our results also show high DC efficacy and shikonin-induced activation and suggest that tail vein injection is likely a sound strategy [46]. Therefore, we considered and expected that the intravenously administered SK-TCL-primed DCs would migrate more rapidly to the lung tissues and then penetrate/reside within the tumor immune microenvironment of the targeted lung organ. However, when the anti-metastatic activities of SK-TCL (Figure 5) and SK-TCL-pulsed DCs (Figure 6) were compared, we observed that the therapeutic effect of SK-TCL treatment was much lower than that of the TCL-pulsed DCs. This limited SK-TCL suppressive effect on tumor metastasis may be due to the immune tolerance of the test TCL sample, which was also administrated through an i.v. injection. Whether other delivery approaches, such as s.c. injection can improve the anti-metastatic activity of TCL will need further study.

Previously, *in vivo* treatment with SK was found to efficiently suppress the skin tissue inflammation [17] and expression of TNF-α [17, 18]. On the other hand, topical treatment with SK was also found to promote EMT and various pro-inflammatory activities, such as increase in expression of MMP2, MMP-9 and vimentin, during wound-healing of skin tissues [20]. In this study, we show that targeting hnRNPA1 with SK may provide a mechanistic explanation for the seemingly contradictory pro- and anti-inflammatory activities of SK at the tissue/organ level. The SK-mediated hnRNPA1 dysfunction may efficiently, but transiently, suppress the splicing and nuclear export activities of specific inflammation-related genes, and this action may result in an interruption of acute cytokine storm. Our current findings on the regulation of hnRNPA1 via SK at a hierarchical and multifaceted level provide deeper mechanistic understanding of the various pharmacological effects of SK reported over the last few decades.

Another plant metabolite, quercetin, has also been found to directly bind to the C-terminal region of hnRNPA1 [60]. However, as compared to the shikonin treatment shown in this study, quercetin treatment was reported to result in a different suppression activity in hnRNPA1-mediated posttranscriptional processes. Quercetin treatment at 100 μM for 18 h, only suppressed the translocation of hnRNPA1 from the cytoplasm back into the nucleus, hence causing accumulation of hnRNPA1 in the cytoplasm [60]. In comparison, SK treatment at 1 μM for 2 h was found in this study to disrupt the hnRNPA1-mediated nuclear export activity of the newly synthesized RNA into the cytosol. These findings together suggest that the disruption mechanisms for hnRNPA1 activity by these two phytochemicals may be different. Although SK is predicted here to interfere with the RNA-binding domain of hnRNPA1, future structural identification of the binding site(s) for SK-hnRNPA1 interaction is still needed to confirm and distinguish the possible binding sites of different phytochemicals on hnRNPA1. Furthermore, as hnRNPA1 has been identified as the target of different plant natural products/metabolites, we therefore consider that hnRNPA1 could be employed as a therapeutic target for other phytochemicals or agents exhibiting anticancer activities. In this regard, the identification of major or specific genes targeted by hnRNPA1 in response to SK or other phytochemical treatments is also an important issue to tackle in order to understand the mechanistic basis of the pharmacological activities of candidate botanical drugs, hopefully leading to their eventual medicinal use.

## MATERIALS AND METHODS

### Compounds

Shikonin (SK) was purchased from Tokyo Chemical Industry (Tokyo, Japan). Doxorubicin (DX), oxaliplatin (OXP) and doceclitaxel (TX) were from Sigma (St. Louis, MO, USA).

### Cell lines and preparation of tumor cell extract

The human breast cancer cell line, MDA-MB-231, was obtained from Dr. Pei-Wen Hsiao (ABRC, Academia Sinica, Taipei) and was originally obtained from American Type Culture Collection (ATCC). MDA-MB-231 cells were grown adherently and maintained in DMEM (Gibco) containing 10% fetal calf serum (FCS), 100 U/ml penicillin and 100 *μ*g/ml streptomycin. Mouse mammary carcinoma 4T1 and 4T1-luc2 (i.e., 4T1 cells transfected by a luciferase cDNA gene) cell lines were also provided by Dr. Pei-Wen Hsiao (Academia Sinica, Taipei, Taiwan) [61]. Both cell types were maintained in RPMI-1640 (Gibco) complete medium (i.e., RPMI-1640 supplemented with 10% FBS, 100 μM non-essential amino acids, 100 μM sodium pyruvate, 100 μg/ml streptomycine and 100 U/ml penicillin). The stably transfected 4T1-luc2 cells were maintained in RPMI-1640 complete medium supplemented with 0.5% puromycin. Cells were grown in a 5% CO_2_ incubator at 37°C. Test samples of various 4T1 tumor cell lysates (TCLs) from phytochemical-treated tumor cells were prepared as described previously [61]. Briefly, after cells were grown to 50% confluence and treated with test compounds, both adherent and floating 4T1 cells were scraped at 24 h or 48 h post-treatment with different test compounds and were centrifuged for 3 min (1,200 rpm). After removing the supernatant, cells were resuspended in PBS and frozen in liquid nitrogen for 1.5 min, then thawed and sonicated for 4 min in a 4°C water bath. The freeze–thaw cycles were then repeated four more times in rapid succession. After the final thaw, cell lysate suspensions were sonicated with three 30-second pulses to further disrupt the aggregates/debris in cell suspension. Prior to use, cell lysates were thawed and centrifuged at 12,000 rpm for 30 min, and the supernatant was then used as the source of tumor antigen. TCL concentrations were determined using the BCA assay (Pierce, Rockford, IL, USA). TCLs were frozen at −80°C until use.

### Molecular docking and virtual screening for predicting interaction affinity between SK and intracellular molecules

The method of *in silico* target fishing has been previously described [62]. In brief, AutoDock 3 (http://autodock.scripps.edu/) with Lamarckian genetic algorithm was used for blind docking of shikonin to protein/peptide structures. A program was created to download all 27317 human protein structures deposited in the Protein Data Bank (http://www.rcsb.org/pdb/home/home.do). The docking results were evaluated using AutoDock-Score. A correlation exists between AutoDock-Score and experimental measured log Kd values [63].

### Antibody-mediated protein pull-down assay

The dynabeads antibody coupling kit (Life Technology; 14311D) was used to pull down intracellular hnRNPA1 molecules in 4T1 or MDA-MB-231 cells according to the manufacturer's recommendations, yielding 10 mg/ml antibody-coupled beads. Briefly, 500 μg TCL in each group were reacted with 2 mg antibody-coupled beads on a roller at RT for 1 h. The precipitated samples were washed 3 times with ddH_2_O and finally dissolved in 100% methanol (30 μl). In each group, the pulled down protein was boiled in methanol for 5 min to denature hnRNPA1 molecules and dissolve out protein-associated SK. These supernatant samples were then assessed by MS/MS analyses for appearance of protein-associated SK molecules. 100% methanol solution was used to reveal the background signal and the pulled down hnRNPA1 molecules from 4T1 cells without SK treatment were used as a negative control for appearance of binding SK.

### Identification of SK using liquid chromatography tandem mass spectrometry

The workflow used for detecting SK using LC-MS/MS has been previously described [28]. In this study, the LC-MS/MS analysis performance was further optimized by coupling an ultrahigh performance liquid chromatography (UPLC) system (ACQUITY UPLC, Waters, Millford, MA) to an ultrahigh resolution tandem mass spectrometer (LTQ Orbitrap Elite, Thermo Fisher Scientific, Bremen, Germany). For LC-MS/MS analysis, solvent A with 20 mM ammonium acetate (pH 9) in the aqueous phase and solvent B with pure ACN were used as the mobile phase for LC separation. The sample was separated by a reverse phase column (HSS T3, 1.8 μm, 2.1 mm × 150 mm, Waters, Milford, MA) at the flow rate of 500 μl/min using gradients of 50-70% solvent B at 0-1 min, 70% solvent B at 1-3 minutes and 70-99% solvent B at 3-5.5 minutes. The total chromatography separation time for each of the analysis was 8 min. In the mass spectrometer, the ion spray voltage was set to −2500 V and the sheath gas and auxiliary (aux) gas flow rate were set to 50 and 15 units, respectively. The capillary temperature and source heater temperature were 360°C and 350°C, respectively. The mass spectrometer was operated in the negative ion mode and set to one full Fourier transform mass spectrometry (FT-MS) scan (m/z 200-300, resolution = 30,000) and one FT-MS selected reaction monitoring (SRM) scan targeted on SK molecule using HCD (ion transition=m/z 287.1 ± 1.0 to m/z 218.02 ± 2.5 with 15,000 product ion resolution). The ion transition for SK was initially determined by the FT-MS product ion scan (m/z 50-300, resolution = 15,000) for precursor m/z of 287.1.

### Expression and purification of human hnRNP A1 (UP1)

The plasmid pET-42(a)-UP1, which allows overexpression of the first 196 amino acids of human hnRNP A1, was constructed by inserting the UP1 fragment into T7 expression vector pET42(a), which included GST and His fusion tag [64]. BL21(DE3) *E. coli* cells carrying the pET-42(a)-UP1 plasmid were grown in LB medium containing kanamycin (30 μg/ml) at 37°C to an OD_600_ between 0.4 and 1.0. Then the temperature was reduced to 20°C and isopropyl β-D-thiogalactopyranoside (IPTG) was added to a final concentration of 1 mM. After 18 h, cells were harvested by centrifugation and stored at −80°C before use.

All purification steps were carried out at 4°C. The frozen cells from 6L culture were thawed and resuspended in 120 ml Buffer A (300 mM NaCl, 50 mM Tris-HCl, 10% glycerol, pH 7.0), and the cells were disrupted by Constant Cell Disruption System. Cell debris was removed by centrifugation and the supernatant was loaded onto a Ni^2+^-NTA column, which was previously equilibrated with buffer A. The column was washed with buffer A, and the GST-His-tagged UP1 was subsequently eluted through a linear imidazole gradient from 30 mM to 500 mM. The fraction of GST-His-tagged UP1 eluted by 100 mM imidazole was collected and reloaded onto a GST column, subsequently eluted through 40 mM reduced glutathione buffer (40 mM reduced glutathione in Buffer A). *The purified* GST-His-tagged UP1 was dialyzed in digestion buffer (100 mM NaCl, 20 mM Tris-HCl, 2 mM CaCl_2_, pH 8.0), followed by cleavage of GST-His fusion tag using Factor Xa protease. After the tag cleavage by Factor Xa, the mixture was loaded onto a Ni^2+^-NTA column and eluted with the Buffer A. The flow-through fraction containing untagged UP1 protein was concentrated and further purified using a *Superdex* 75 10/300 *gel filtration column. The resulting eluent was concentrated and sorted at −80*°C before use.

### Isothermal titration calorimetry analysis

Prior to the isothermal titration calorimetry (ITC) test, the hnRNPA1 protein, SK and RNA (WT) samples were prepared into an identical concentration of the dialysis buffer and DMSO (0.1%). The dialysis buffer contained 300 mM NaCl, 50 mM Tris-HCL and 10% glycerol. All ITC runs were carried out on a MicroCal iTC200 instrument. Protein concentration in the sample cell was 14 μM, and SK in the titrant syringe was at 300 μM. For displacement titration analysis, protein, WT and SK were made at concentrations of 3, 60 and 60 μM, respectively. Samples in the syringe were injected at a volume of 2 μL (with a total of 18 injections) for every ITC test. There was a 120 second spacing time between each injection, with a stir speed of 1000 rpm. The amount of heat (microcal) was plotted against the injection number to give the raw data, as shown by peaks corresponding to each injection. The obtained raw data peaks were converted using the Origin7 software to make a plot of the enthalpy change per mole of injectant (ΔH, kcal mol^−1^) against molar ratio.

### Electrophoretic mobility shift assay

The gel shift assay was performed by using biotin-5′-end-labeled DNA (TR2: TTAGGGTTAGCG) or RNA (WT: 5′-AUUUAGGGAUUUAGGGAAUG-3′′; 5′-AUUUCGGGAUUUCGGGAAUG-3′) nucleotide chains. The purified recombinant UP1 was incubated with increasing amounts of shikonin for 30 min. Then the mixture was incubated with the biotin-5′-end-labeled RNAs in binding buffer (20 mM HEPES, 50 mM KCl, 1 mM EDTA, 5 mM MgCl_2_, 0.1 mg/ml tRNA, 0.1 mg/ml BSA, 5 mM DTT, 10% glycerol) at room temperature for 30 min. The samples were loaded on a 5% native polyacrylamide gel and run at 150 V for 25 min at room temperature, and then transferred to nylon membrane at 100 V for 10 min. When transfer was complete, RNAs and membrane were cross-linked at 120 mJ/cm^2^ by using a UV cross-linker. The UP1-RNAs complex was detected by *LightShift* Chemiluminescent *EMSA* Kit following the manufacturer's instructions.

### Mice

For this study, female BALB/c mice aged 6-8 weeks were purchased from the National Laboratory Animal Breeding and Research Center, Taipei, Taiwan. Test mice were maintained in a laminar airflow cabinet kept at 24 ± 2°C and 40-70% humidity with 12-h light/12-h dark cycles under specific pathogen-free conditions. All manipulation and experimental protocols involving animals were approved by the Institutional Animal Care and Utilization Committee (IACUC) of Academia Sinica, Taipei.

### 4T1 mammary carcinoma-tumor resection model

Mice were injected subcutaneously with 4T1-Luc2 cells (5 × 10^5^/100 μl PBS/mouse) into the fourth mammary fat pad under isoflurane anesthesia. Tumor growth was monitored by measuring the tumor volume according to the formula: volume = length × (width)^2^/2. After tumors were established (180-200 mm^3^) on day 15, test mice were divided into different groups (8 mice/group) and subjected to different treatments. At 15 days post tumor cell implantation, primary 4T1 tumors *in situ* were surgically removed by a tumor resection process. For immunization with test TCL vaccines, mice were then administered with control (PBS) or specific TCL (100 μg/200 μl/mouse) by intravenous injection for 3 weeks (3 injections/week) post tumor resection. To monitor progression of metastatic tumors, control (PBS injection only) and different TCL-treated mice were compared for their tumor metastatic activity and survival rate in another 8 weeks. For immunization with DC vaccines, mice were administered with specific or control DC vaccines (1 × 10^6^ DCs/200 μl PBS/mouse) by intravenous injection at 1, 8 and 15 days post tumor resection. Bioluminescence signals from the 4T1-luc2 tumor cells in test mice were analyzed using a non-invasive IVIS imaging system (Calipers, Hopkinton, MA) after intraperitoneal injection of 150 mg/kg D-luciferin (NanoLight Technology, Pinetop, AZ).

### Expression of hnRNPA1 by lentivirus transfection system

To produce lentiviruses containing human or mouse hnRNPA1 expression plasmid (pLKO.AS2 expression vector containing human or mouse hnRNP A1) or the control plasmid, 1.5 × 10^7^ 293T cells were seeded in a 15 cm dish and incubated (37°C, 5% CO_2_) overnight. On day 2, the medium was changed without serum freshly 60 minutes before transfection. PEI solution and viral packaging plasmids (pCMV-ΔR8.91, pMD.G and pLKO.AS2) were diluted individually into serum-free medium and incubated at room temperature for 10 minutes. The diluted PEI solution was added into the plasmid mixture and mixed well by pipetting. The mixture was then incubated at room temperature for 10 minutes. The entire content of the tube was pipetted into a 293T culture dish, and mixed gently by tilting the plate back and forth. After 6 h, 1.5 ml FBS was added into the culture medium. The cells were then incubated for 16 h. At day 3, the media containing lentivirus was harvested and stored at 4°C. Then the media was replaced with media containing 10% FBS per dish. The cells were then incubated for 24 h. On day 4, the same procedure as conducted on day 3 was repeated. On day 5, the media containing lentivirus was harvested. All the harvested media were then pooled and the cells were spun down at 1200 rpm for 5 minutes. The supernatant was then transferred to a syringe and further filtered by a 0.45 μm syringe filter. The flow-through was transferred to centrifuge tubes and further centrifuged by ultra-centrifuge at 25000 rpm for 2 h. Then the supernatant was removed and the lentivirus was resuspended with TNF buffer (10 mM Tris-HCl, 100 mM NaCl, 1 mM EDTA, PH 7.4) by adding the pellet. The virus was left at 4°C overnight to complete the resuspension.

### Mouse bone marrow-derived dendritic cells

Mouse bone marrow-derived dendritic cells (BMDCs) were generated and modified as previously described [65]. Briefly, bone marrow tissues were collected from BALB/c mice, and erythrocytes were removed. The derived bone marrow cells were cultured in complete RPMI 1640 medium supplemented with 20 ng/mL GM-CSF and 50 μM 2-mercaptoethanol. On day 2, two-thirds of the original medium was replaced by 30 mL fresh medium. On day 5, the floating cells were gently removed and the culture replenished again with fresh medium containing 20 ng/mL GM-CSF and 20 ng/mL IL-4. On day 7, the nonadherent and loosely adherent DCs in culture were harvested and used as the source of dendritic cells in making various test vaccines. DCs generated in this manner were mainly immature DCs (85% of cells are CD11c^+^) and displayed the typical morphologic features of DCs.[66]

### Activation of tumor cell lysate-pulsed DCs

Activation of bone marrow-derived DCs (BMDCs) was performed as previously described [66]. Briefly, BMDCs were incubated for 2 h with various test tumor cell lysate (TCL) samples containing 200 μg protein/ml. LPS (1 μg/ml) was then added to the culture medium for co-cultivation with TCL-loaded DCs for another 22 h. DCs treated with LPS (mDCs) or in combination with test preparations were then used as vaccinated DCs. DC samples reacted with naïve-TCLs (i.e., cell lysates collected from 4T1 tumor cells that were treated without SK stimulation) were designated as naïve-TCLs-pulsed DCs. DC samples treated with SK-TCLs, obtained from 4T1 cells treated with 1 μM SK for 24 h, were designated as SK-TCL-pulsed DCs. Similarly, DCs reacted with SK-4T1(hn)-TCL, obtained from 4T1-hnRNPA1 cells treated with 1 μM SK, were designated as SK-4T1(hn)-TCL-pulsed DCs. After treatment with various TCLs, vaccinated DCs were washed with PBS 3 times and each DC vaccine preparation (1 × 10^6^ DCs/administration) was compared for their anti-metastatic effect on 4T1 tumors *in vivo*.

### Western blot assay

Tumor cell lysate samples were prepared as previously described [8, 66]. To assay for expression of ICD-associated markers, 4T1 TCL protein samples were resolved by SDS PAGE using 8, 10 or 15% stepwise gels. The resolved proteins were transferred onto a PVDF membrane (Novex, San Diego, CA) and blotted with anti-hnRNPA1 (rabbit polyclonal; GeneTex), anti-HMGB1 (rabbit plyoclonal; GeneTex), anti-HSP70 (rabbit plyoclonal; GeneTex), anti-CRT (rabbit polyclonal; Abcam), or anti-β-actin (rabbit polyclonal; Abcam). The membrane was blocked with 5% non-fat dry milk in PBST buffer [phosphate-buffered saline (PBS) containing 0.1% Tween 20] for 60 min at room temperature. Blotted membranes were then incubated overnight at 4°C with specific, commercially available antibodies (1:1,000 dilutions). Loading of equal amounts of protein was assessed using mouse β-actin protein as a reference. The blots were rinsed three times with PBST buffer for 5 min each. Washed blots were incubated with HRP-conjugated secondary antibody (goat polyclonal; Abcam; 1:100,000 dilution) and washed again three times with PBST buffer. The transferred proteins were visualized with an enhanced chemiluminescence (ECL) detection kit (Amersham Pharmacia Biotech, Buckinghamshire).

### Immunofluorescence staining

4T1, 4T1-hnRNPA1, MDA-MD-231 or MDA-MD-231-hnRNPA1 cells were initially seeded onto glass coverslips (Deckglaser) at 5 × 10^4^ cells/ml in 24 well plates overnight. After SK or Dox treatment, immunofluorescence was performed on cells fixed in 4% paraformaldehyde and stained with anti-CRT (rabbit polyclonal; Abcam) or anti-BrDU/BrU (rabbit polyclonal; Abcam) antibodies followed by indirect immunofuorescence using Donkey Anti-Rabbit IgG H&L antibody (Alexa Fluor 488; Abcam). Each coverslip was then inverted onto a slide containing 10 μl of DAPI-containing mounting solution (SouthernBiotech). Fluorescence imaging was performed and captured on a LSM 780 inverted Confocal plus Super Resolution Microscope (Zeiss).

### BrU Incorporation

4T1, 4T1-hnRNPA1, MDA-MD-231 or MDA-MD-231-hnRNPA1 cells were grown overnight on coverslips, washed 3 times with HBSS and placed in 100 μl cell buffer containing 2 mM bromouridine (Sigma). Cells were incubated for 120 min at room temperature after SK treatment. The medium was aspirated, and cells were fixed with 2% paraformaldehyde and stained by anti-BrU antibody for immunofluorescence staining.

### MTT assay

4T1, 4T1-hnRNPA1, MDA-MD-231 or MDA-MD-231-hnRNPA1 cells (1 × 10^5^cells/ml) dispensed in 96-well plates were incubated with vehicle or test compounds for 24 h in each basal medium in a 5% CO_2_ incubator. All treatments were performed in triplicate cultures. The cytotoxic effect of SK and Dox on test tumor cells were assayed using the 3-(4, 5-dimethythiazol-2-yl)-2, 5-diphenyl tetrazolium bromide (MTT) colorimetric assay. The absorbance at 570 nm (A570) was measured by a multiwall scanning spectrophotometer.

### RT-PCR

Total RNA was isolated from drug-treated MDA-MB231/MDA-MB231-hnRNP A1 cells by using TRIzol reagent (Invitrogen), and then reverse transcribed to cDNA (SuperScript III Reverse Transcriptase Kit, Invitrogen). The cDNA samples were then analyzed by RT-PCR with primers designed to bind to PKM1 (forward primer: 5′-TGTGCGAGCCTCAAGTCAC TCCACA-3′ and reverse primer: 5′-TCGGGCCTTGCCAACATTCATGGCAAA-3′), PKM2 (forward primer: 5′-CCGCCGCCTGGCGCCCATTACCAGC-3′ and reverse primer: 5′-CTGT CTGGGGATTCCGGTCACAGCAATGATG-3′), and both *Ron* and *ΔRon* (forward primer: 5′-CCTGAATATGTGGTCCGAGACCCCCAG-3′ and reverse primer: 5′-CTAGCTGCTTCC TCC GCCACCAGTA-3′) [37].

### hnRNPA1 siRNA treatment

4T1 cells were seeded in a 6-well plate at 5 × 10^5^ cells/well for 24 h before transfection. siRNAs used for knockdown of mouse hnRNPA1 were purchased from Biotools (Taiwan, ROC) as follows: hnRNPA1 siRNA sequence: (Hnrnpa1-siRNA-3: 5′-AUUAGGAAUAUUAUGGGCCTT-3′); negative control siRNA (Neg; sequence: 5′-ACGUGACACGUUCGGAGAATT-3′). At the beginning of transfection, hnRNPA1 siRNA (100 pmol) was diluted in 250 μl Opti-MEM I Reduced Serum Medium. Aliquots of 5 μl Lipofectamine 2000 transfection reagent (Invitrogen) were diluted with 250 μl Opti-MEM I Reduced Serum Medium. Diluted siRNA were mixed gently with the diluted Lipofectamine 2000 and incubated for 20 min at room temperature. The siRNA-Lipofectamine 2000 complexes were subsequently added to each well containing cells and medium. Cells were incubated at 37°C for 48 h until test cells were ready to be treated with test chemicals.

### Statistical analysis

For RT-PCR analysis, P values for comparing the expression ratio of *PKM1*/*PKM2* and Δ*RON*/*RON* were calculated using Student's *t* test. For animal experiments, 8 mice were assigned per treatment group. Data were analyzed by two-way ANOVA. Statistical analyses were conducted with GraphPad Prism 5.0 (San Diego, CA). Differences in mouse survival time and rate were determined by a log-rank (Mantel-Cox) test of the Kaplan-Meier survival curves. All statistical tests were two-sided (*, *P* < 0.05; **, *P* < 0.01; n.s, no significance).

## SUPPLEMENTARY FIGURE



## Abbreviations

CRT: calreticulin
DAMPs: damage-associated molecular patterns
EMT: epithelial-to-mesenchymal transition
HMGB1: high-mobility group box 1 protein
hnRNPA1: heterogeneous nuclear ribonucleoprotein A1
ICD: immunogenic cell death
ITC: isothermal titration calorimetry
MDSC: myeloid-derived suppressor cell
PKM2: pyruvate kinase-M2
SK: shikonin
TCL: tumor cell lysate
TNF-α: tumor necrosis factor-α
