# Correction: Immunogenicity of mammary tumor cells can be induced by shikonin via direct binding-interference with hnRNPA1

**DOI:** 10.18632/oncotarget.26007

**Published:** 2018-08-14

**Authors:** Shu-Yi Yin, Thomas Efferth, Feng-Yin Jian, Yung-Hsiang Chen, Chia-I Liu, Andrew H.J. Wang, Yet-Ran Chen, Pei-Wen Hsiao, Ning-Sun Yang

**Affiliations:** ^1^ Agricultural Biotechnology Research Center, Academia Sinica, Taipei, Taiwan, ROC; ^2^ Institute of Pharmacy and Biochemistry, University of Mainz, Germany; ^3^ School of Medical Laboratory Science and Biotechnology, Taipei Medical University, Taipei, Taiwan, ROC; ^4^ Institute of Biological Chemistry, Academia Sinica, Taipei, Taiwan, ROC

**This article has been corrected:** An institutional investigation was conducted at the request of the Oncotarget editorial staff by the Academia Sinica Ethics Committee (Taipei, Taiwan). The Committee concluded the following: “The partial duplication of Figure 4a and 4b is judged to be a mistake during data processing, rather than a research misconduct.” The correct Figure 4B is shown below; 4A appears correctly in the original article. The authors declare that these corrections do not change the results or conclusions of this paper.

**Figure 4:**
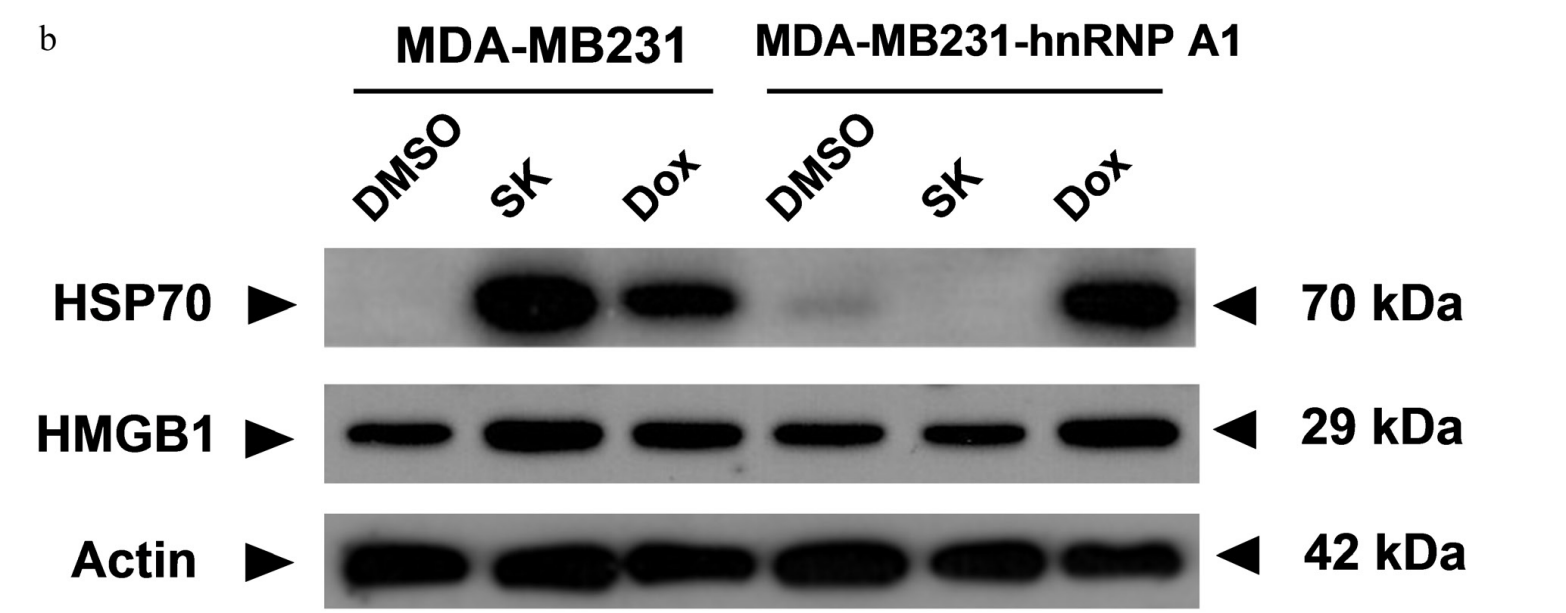
hnRNPA1 is a critical mediator of the SK-induced ICD activity in mammary tumor cells **a.** and **b.** Western blot analyses of expression of HSP70 and HMGB1 in human (MDA-MB-231) and mouse (4T1) tumor cells. Some 4T1, 4T1-hnRNPA1, MDA-MB-231 and MB-231-hnRNPA1 cells were treated with SK or Dox at 5 μg/ml for 24 h. β-actin was used as a loading control.

Original article: Oncotarget. 2016; 7:43629-43653. https://doi.org/10.18632/oncotarget.9660

