# Supplementary material for: Immunogenicity of mammary tumor cells can be induced by shikonin via direct binding-interference with hnRNPA1
Source: Oncotarget. 2016 May 27;7(28):43629–53. doi: 10.18632/oncotarget.9660 (PMC5190049; doi:10.18632/oncotarget.9660)
Supplement: Supplementary file 1 [file oncotarget-07-43629-s001.pdf]

Immunogenicity of mammary tumor cells can be induced by shikonin via direct binding-interference with hnRNPA1

SUPPLEMENTARY FIGURE

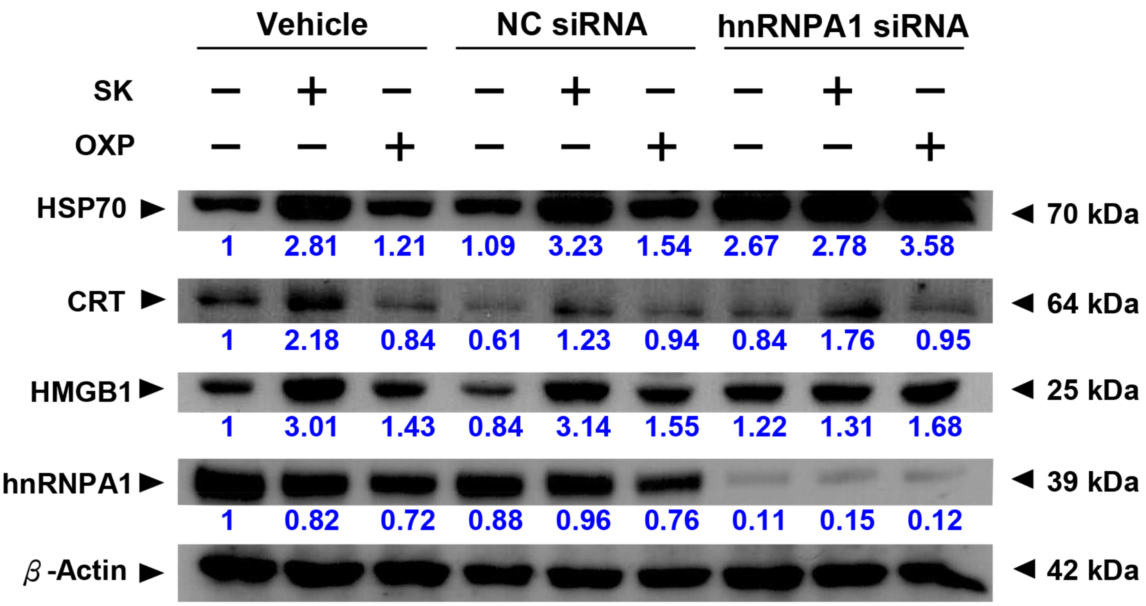

Supplementary Figure S1: Western blot analyses of expression of ICD markers, including HSP70, CRT and HMGB1, on 4T1 mammary tumor cells. Test 4T1 cells were dispensed in 6-well plates (3×10^5 cells/well) and incubated with vehicle, 5 μM shikonin (SK) or 200 μM oxaliplatin (OXP) for 24 h. To evaluate the role of hnRNPA1 in SK- and OXP-induced exposure of ICD features, knockdown of mouse hnRNPA1 expression in 4T1 cells was performed with designed siRNAi treatment. The negative control (Neg) RNAi was used as a nonspecific control. Beta-actin was used as a loading control. The protein expression levels were quantified using ImageJ software. Fold-changes of expression level were normalized to the value in untreated 4T1 cells of the vehicle group and were indicated by the number labeled in blue. Similar results were obtained from three independent experiments.
